# Mobile intraoperative CT-assisted frameless stereotactic biopsies achieved single-millimeter trajectory accuracy for deep-seated brain lesions in a sample of 7 patients

**DOI:** 10.1186/s12883-021-02322-5

**Published:** 2021-07-22

**Authors:** Oliver Bichsel, Markus F. Oertel, Lennart H. Stieglitz

**Affiliations:** 1 Department of Neurosurgery, University Hospital Zurich, University of Zurich, Zurich, Switzerland; 2 Clinical Neuroscience Center, University Hospital Zurich, University of Zurich, Zurich, Switzerland

**Keywords:** Stereotactic neurosurgery, Image-guidance, Intraoperative CT, Brain biopsy

## Abstract

**Background:**

Brain biopsies are crucial diagnostic interventions, providing valuable information for treatment and prognosis, but largely depend on a high accuracy and precision. We hypothesized that through the combination of neuronavigation-based frameless stereotaxy and MRI-guided trajectory planning with intraoperative CT examination using a mobile unit, one can achieve a seamlessly integrated approach yielding optimal target accuracy.

**Methods:**

We analyzed a total of 7 stereotactic biopsy trajectories for a variety of deep-seated locations and different patient positions. After rigid head fixation, an intraoperative pre-procedural scan using a mobile CT unit was performed for automatic image fusion with the planning MRI images and a peri-procedural scan with the biopsy cannula in situ for verification of the definite target position. We then evaluated the radial trajectory error.

**Results:**

Intraoperative scanning, surgery, computerized merging of MRI and CT images as well as trajectory planning were feasible without difficulties and safe in all cases. We achieved a radial trajectory deviation of 0.97 ± 0.39 mm at a trajectory length of 60 ± 12.3 mm (mean ± standard deviation). Repositioning of the biopsy cannula due to inaccurate targeting was not required.

**Conclusion:**

Intraoperative verification using a mobile CT unit in combination with frameless neuronavigation-guided stereotaxy and pre-operative MRI-based trajectory planning was feasible, safe and highly accurate. The setting enabled single-millimeter accuracy for deep-seated brain lesions and direct detection of intraoperative complications, did not depend on a dedicated operating room and was seamlessly integrated into common stereotactic procedures.

## Introduction

Stereotactic biopsies are essential neurosurgical procedures. With the least possible invasiveness and morbidity, they enable sampling of central nervous system lesions for histopathological diagnosis, thereby providing valuable information for treatment and prognosis. Through the advent of advanced neuroimaging methods and stereotactic techniques, biopsies can now be performed minimally-invasive and with a good diagnostic yield [14]. The efficacy of stereotactic biopsies is mainly dependent on the accuracy and precision of biopsy cannula placement with the frontrunner for brain biopsy currently being frame-based stereotaxy [14].

Since the surge of techniques that allow localization of histopathological probes through real-time intraoperative imaging, formerly blind stereotactic approaches have been transformed into visually controlled, highly accurate procedures. A decisive advantage of intraoperative probe localization is that it allows trajectory modifications during surgery, thereby eliminating misdiagnosis secondary to faulty targeting or brain-shift. A further benefit is that intraoperative imaging can immediately visualize complications, foremost hemorrhages. Especially for deep-seated intracerebral lesions, intraoperative image-guided biopsy techniques have been shown to be superior to other techniques [2, 3].

Despite the known superiority of real-time volumetric intraoperative imaging using MRI [11, 15] for the localization of probe targets, stereotactic X-ray and fluoroscopy are still in common use [20]. Two-dimensional radiographic imaging methods, however, provide only limited positional information and repetitive avoidable irradiation is necessary in order to achieve perfect alignment during the procedure [4]. Moreover, the sensibility for detecting intraoperative complications is limited.

An alternative would be a mobile CT unit, that has already been investigated for the verification of stereotactically implanted deep brain stimulation electrodes [4–6, 16], showing accurate intraoperative three-dimensional confirmation of the electrode position, or to confirm the applicator position in laser interstitial thermal therapy [12]. Mobile CT units do not depend on dedicated operating rooms and can be easily incorporated into the procedure without disrupting the surgical workflow as no patient transport or repositioning is required. Compared to intraoperative MRI, mobile CT units are less time-consuming and cost-intensive. Recently, the utilization of a mobile CT unit for stereotactic biopsies has been investigated and showed a significantly shorter intervention time [8]. However, the study does not report trajectory accuracies and relies on a frame-based approach. In contrast, frameless approaches have the potential to reduce intervention times while having an equivalent diagnostic yield, morbidity and mortality [7].

We hypothesize that the combination of a mobile CT unit with stereotactic frameless biopsy procedures could provide optimal outcome regarding accuracy, precision and operating time. Together with prior careful trajectory planning using MRI data, complications can be reduced by consideration of cortical vasculature as well as sulcal-pial boundaries [1, 19]. Merging preoperative MRI and pre-procedural intraoperative CT images has the additional advantage of reducing shifting between the brain and instruments. In the literature, reported accuracy measures typically range between one to a few mm [13, 20].

To our knowledge this is the first report of a mobile intraoperative CT unit to assist and verify frameless stereotactic biopsy trajectories and procedures. Our goal was to assess the incorporation of the mobile intraoperative CT unit into neurosurgical biopsies, to describe the method in detail and to determine the safety and accuracy of this approach.

## Materials and methods

**Fig. 1:**
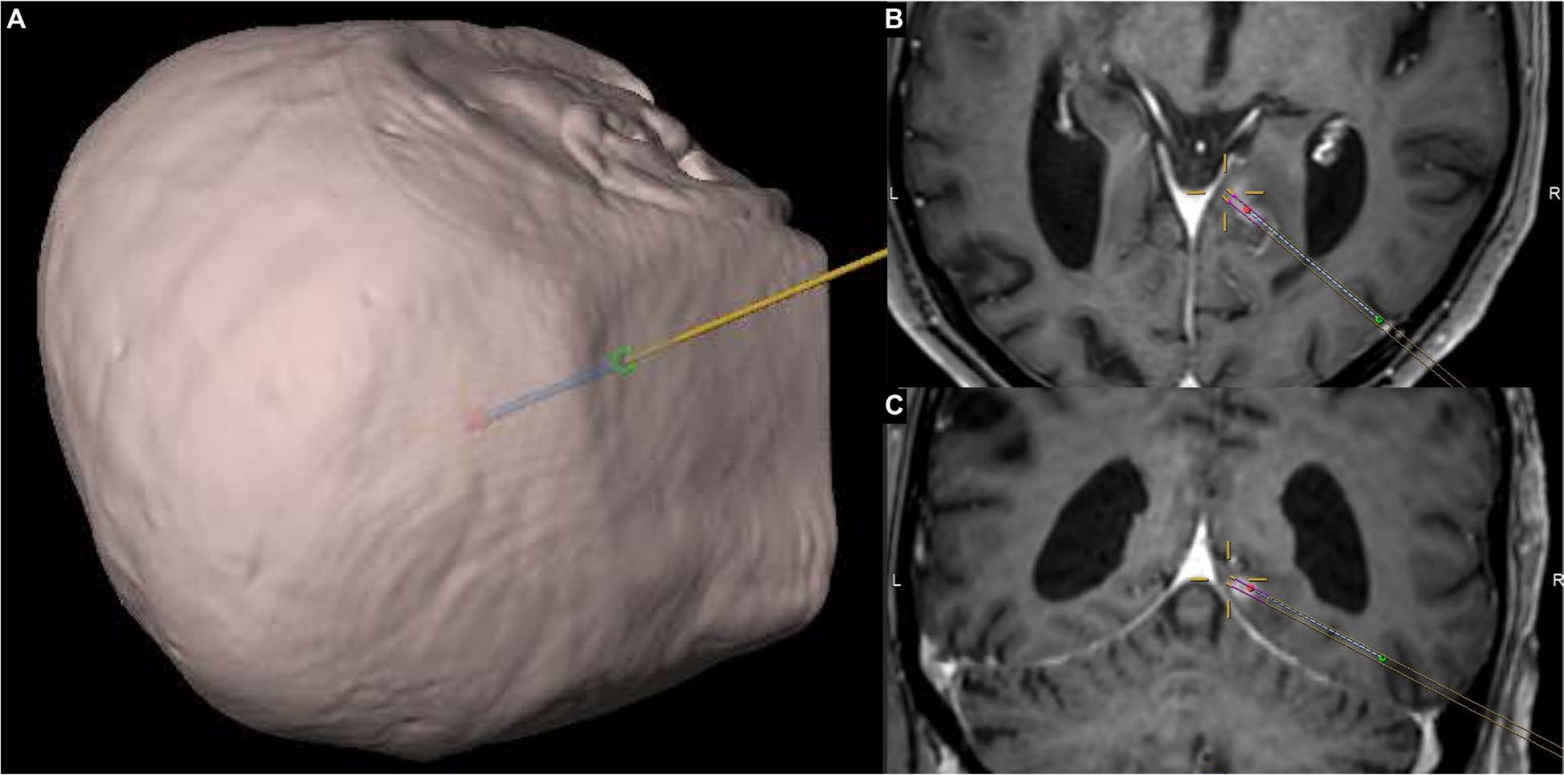
Stereotactic planning and cannula position verification in an exemplary patient. **A** three-dimensional image-based head reconstruction with planned biopsy trajectory from a right occipital approach. **B** axial and **C** coronal T1-weighted contrast-enhanced MRI sequences. The target is indicated by the crosshairs; mainly out of plane are the planned trajectory (parallel lines), the planned biopsy region (violet), as well as the intra-procedural CT-verified tip, intracranial trajectory and entry point of the biopsy cannula (red, dashed line and green point, respectively)

A total of 7 stereotactic trajectories in 7 patients have been verified intraoperatively using a mobile CT unit (Table 1). This study was approved by the local ethics committee (Kantonale Ethikkommission Zürich, BASEC-Nr.: 2020–00,810). For all patients, thin-cut (slice thickness of at most 1 mm) T1-weighted sequences with contrast were used for stereotactic trajectory planning with the software iPlan® (Brainlab, Feldkirchen, Germany). For ID 5 a PET-MR and for IDs 1, 2 and 6, additional FLAIR sequences were fused for preoperative planning. For the operation, patients were positioned depending on the planned trajectory and site of lesion, with their head fixated in a DORO® radiolucent headrest (ProMed Instruments, Freiburg, Germany). An intraoperative, pre-procedural CT examination was obtained using the Airo® mobile CT (Brainlab, Feldkirchen, Germany) with fixed collimator and reconstruction slice width (1.06 mm and 1.0 mm, respectively) and a kVp of 120. mAs was 325 for IDs 1, 4, 7 and 260 for all others. The scan was automatically stereo-localized by the Brainlab Curve neuronavigation system (Brainlab, Feldkirchen, Germany) and computationally fused with the pre-operative MRI images. Stereotactic Nashold-type disposable brain cannula (Brainlab, Feldkirchen, Germany) biopsy was performed using the VarioGuide® (Brainlab, Feldkirchen, Germany) frameless stereotactic guidance tool. Specimen were extracted for frozen sections and final histological analysis with routine and immunohistochemical studies as indicated. While waiting for the results of the frozen sections, an intra-procedural CT was obtained using the Airo® unit to verify the correct position of the *in-situ* biopsy cannula (Fig. 1). Finally, the cannula was removed and surgery finished.

**Table 1:** Characteristics of patients, lesions and procedures. Operation duration was measured from incision to suture

Patient number	Age-range [years]	Localisation	Largest diameter [mm]	Operation duration [min]	Position	Histology
1	20–29	Pineal	20	130	Prone	Germinoma
2	70–79	Thalamic	23	150	Supine	High-grade glioma
3	60–69	Crus cerebri	18	53	Supine	High-grade glioma
4	70–79	Gyrus cinguli	13	115	Semi-lateral	High-grade glioma
5	60–69	Trigonal	17	86	Supine	High-grade glioma
6	80–89	Pons	29	115	Semi-lateral	B-cell lymphoma
7	50–59	Foramen of Monro	25	105	Supine	High-grade glioma

### Accuracy assessment

The anterior commissure (AC) and the posterior commissure (PC) were manually determined according to the iPlan® software in the pre-operative MRIs in order to define a comparable coordinate system. The coordinates of two points along the visualized biopsy cannula (tip and entry point of cannula) were exported. The radial trajectory deviation (*d*) was calculated as the shortest distance from the target point (${x}_{0}$) to the infinite straight line connecting the entry point and the tip of the cannula (${x}_{1}$ and ${x}_{2}$; $d=\frac{\left|({x}_{0}-{x}_{1})\times ({x}_{0}-{x}_{2})\right|}{\left|{x}_{2}-{x}_{1}\right|}$). The mean radial trajectory accuracy was then obtained by averaging the radial trajectory deviation of each procedure. We focused on the radial trajectory deviation as a measure of accuracy, and not for instance the Euclidean error vector, as the depth of the cannula along an accurate trajectory can be changed with relative ease when an intraoperative image is available. The biopsy distance was calculated as the length of the line between the coordinates of the entry point and the tip of the cannula.

## Results

### Clinical results

We included patients with a wide range of different lesion locations as well as operating positions (Table 1). Relocation of the biopsy cannula due to off-target position of the cannula or negative frozen sections was not required. Our technique was safe in all cases with no perioperative complications. The volume CT dose index (CTDI_vol_) per scan was 38.8 mGy in four patients and 64.7 mGy in the other three patients. Postoperative control imaging was only performed in the patient with the Foramen of Monro mass lesion on the first day after surgery to exclude hydrocephalus.

### Accuracy

**Fig. 2:**
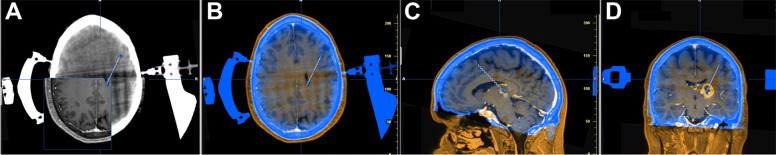
Computational merging of a planning MRI and the pre-procedural CT with the headrest in place in an exemplary patient. **A** Pre-procedural CT-scan with an inlet of the merged planning MRI. **B** axial, **C** sagittal and **D** coronal view of the planning T1-weighted contrast-enhanced MRI sequences (orange) with the merged native CT images (blue)

Intraoperative imaging for pre-procedural image merging (Fig. 2) as well as peri-procedural to verify the position of the biopsy cannula was successful in all patients. The mean radial trajectory deviation was 0.97 ± 0.39 mm at a biopsy depth of 60 ± 12.3 mm (mean ± standard deviation, Table 2). The interobserver (LS and OB) variability of the radial trajectory deviation was assessed in 4 patients and was below 0.1 mm.

**Table 2:** Trajectory lengths and radial trajectory deviations of all biopsies. *SD* standard deviation

Patient Number	Trajectory length [mm]	Radial trajectory deviation [mm]
1	66.8	0.7
2	67.6	1.4
3	75.6	1.2
4	49.5	0.8
5	67.8	0.8
6	37.2	1.5
7	55.4	0.4
Mean ± SD	60 ± 12.3	0.97 ± 0.39

## Discussion

Biopsies of central nervous system lesions are a common and crucial task for neurosurgeons, providing highly relevant information for treatment and prognosis. While a plethora of different approaches to sampling brain lesions exist, their diagnostic yield is largely depending on a methods’ accuracy and precision. Whereas an accuracy of a few mm might be acceptable for superficial and large lesions, more elaborated approaches are required for deep-seated and small lesions. Here, we present an optimized workflow with which we have achieved single millimeter-accuracy for deep-seated brain lesions by using a mobile CT unit in combination with neuronavigation-based frameless stereotaxy and pre-operative MRI-based trajectory planning.

### Single-millimeter accuracy for deep-seated lesions

In our study, most of the lesions were located at a long distance from the brain surface with a mean value from the cortex surface to the intracerebral lesion of 6 cm and an upper range of up to 7.6 cm. For these deep-seated locations, our mobile unit CT-assisted biopsy technique was able to reach a single-millimeter accuracy. This highly accurate approach especially motivates its use for very small, deep-seated lesions or lesions in highly eloquent areas, such as the brainstem (Patient 3 and 6) or thalamus (Patient 2).

### Seamlessly integrated intraoperative cannula position verification

Intraoperative biopsy cannula position verification reduces the possibility of futile biopsies due to false targeting and considers brain-shifts. While intraoperative MRI or conventional CT scans can also provide biopsy cannula localization, they are time-consuming, costly, require a dedicated room and amagnetic instruments (in the case of intraoperative MRI) or largely disrupt surgery due to limited patient positions, repositioning or transfer. Our mobile CT unit was able to verify biopsy cannula positions without depending on a dedicated operating room and without relevantly interfering with the surgical workflow. There were no limitations as to the lesion locations or patient positions as the mobile CT unit could be transferred directly to the operating table. Such a seamless integration can also be provided with plain x-rays or C-arms, although only with the limitations of two-dimensional imaging and lower image quality. One work group using the C-arm reported a mean target error of 1.44 ± 1.43 mm [20]. We focused on the radial trajectory deviation as a measure of accuracy, as the depth of the cannula along an accurate trajectory can be modified with relative ease when an intraoperative image is available, and achieved a radial trajectory accuracy of 0.97 ± 0.39 mm using our frameless approach with a mobile CT unit. Our mean operation time (incision to suture) of 108 min (standard deviation of 31 min) is in the range of other frameless and frame-based approaches [8, 10, 14, 17, 18], and is of importance as avoiding disruptions of surgery or patient transfers and shorter procedure times relate with shorter durations of anesthesia and risk of infections. This total operation time included a minimum of 20 min period of waiting for the frozen section results, which we could efficiently use for the second intraoperative CT scan in order to verify the cannula position. Furthermore, compared to frame-based approaches, a frame-less approach allows a more flexible and larger area of trajectory entry points (especially for posterior fossa lesions and trajectory entries close to the frame), and might even allow the incorporation of robotic stereotactic systems [9].

### Eliminating relative shifts between the planned trajectory and stereotactic instruments

Preoperative MRI-planned trajectories were transferred to intraoperative CT images through computational intermodal image merging. Using a frameless real-time-imaging approach, we also eliminated the possibility for relative shifts between the brain and our stereotactic instruments, thereby further increasing targeting accuracy. Thanks to meticulous trajectory planning using MRI data and our single-millimeter accuracy, our technique was safe in all cases, with no perioperative complications. Furthermore, relocation of the biopsy cannula due to off-target positioning of the cannula or negative frozen sections was never required.

### Limitations

The generalizability of this study is limited by a small cohort size. Nevertheless, given our small variance in radial trajectory deviation, the highly accurate mobile intraoperative CT-assisted approach for frameless stereotactic biopsies of deep seated lesions clearly prompts its further use and investigation in a larger cohort.

## Conclusion

We could demonstrate a single-millimeter accuracy for neurosurgical biopsies of deep-seated lesions assisted by a mobile intraoperative CT unit, which was seamlessly integrated in our frameless, MRI-planned stereotactic procedure and operative workflow. Routine application of our technique could reduce the risk of inconclusive diagnosis and reoperations due to inaccurate or faulty targeting, confirm cannula and biopsy position and provide immediate imaging of complications.

## Data Availability

The datasets generated and analysed during the current study are not publicly available due personal health information protection regulations but are available from the corresponding author on reasonable request.
